# Differentiation of multiple myeloma and metastases: Use of axial diffusion-weighted MR imaging in addition to standard MR imaging at 3T

**DOI:** 10.1371/journal.pone.0208860

**Published:** 2018-12-17

**Authors:** Ga Eun Park, Won-Hee Jee, So-Yeon Lee, Jin-Kyeong Sung, Joon-Yong Jung, Robert Grimm, Yohan Son, Mun Young Paek, Chang-Kee Min, Kee-Yong Ha

**Affiliations:** 1 Department of Radiology, The Catholic University of Korea, Seocho-gu, Seoul, South Korea; 2 Department of Radiology, KangbukSamsung Hospital, Sungkyunkwan University School of Medicine, Jongno-gu, Seoul, South Korea; 3 Siemens Healthcare, Erlangen, Germany; 4 Siemens Healthcare Ltd. Poongsan Building, Seodaemun-gu, Seoul, South Korea; 5 Department of Internal Medicine, The Catholic University of Korea, Seocho-gu, Seoul, South Korea; 6 Department of Orthopedic Surgery, The Catholic University of Korea, Seocho-gu, Seoul, South Korea; George Washington University, UNITED STATES

## Abstract

**Background:**

Metastasis and multiple myeloma are common malignant bone marrow lesions which may be difficult to distinguish because of similar imaging findings. The purpose of this study was to determine the value of adding diffusion-weighted imaging (DWI) to standard MR imaging to differentiate multiple myeloma from metastasis.

**Methods:**

25 patients with metastasis and 18 patients with multiple myeloma underwent 3T MR imaging with DWI (b = 0, 800 s/mm^2^) were enrolled. They all had pathologically confirmed bone lesions and were in a treatment naïve state. Two readers who were blind of final diagnosis measured the average ADC (ADCav) and minimum ADC (ADCmin) on the DWI. They then estimated the diagnosis, based on the standard MR imaging and measured ADC values. Another reader performed histogram analysis on the whole tumor volume and obtained mean ADC (ADCvol), standard deviation (SDvol), skewness, and kurtosis. Comparison of the obtained values from DWI was performed by the t-test or Mann-Whitney U test. The receiver operating characteristic (ROC) curve with areas under the curve (AUC) was used to obtain the cut off values and to evaluate the diagnostic performance of the two readers.

**Results:**

ADCav, ADCmin, and ADCvol of multiple myeloma were significantly lower than those of metastasis: ADCav, 752 μm^2^/sec versus 1081 μm^2^/sec; ADCmin, 704 μm^2^/sec vs 835 μm^2^/sec; ADCvol 761 μm^2^/sec vs 1184 μm^2^/sec (*p* < .001). In histogram analysis, ADC values of multiple myeloma showed narrow distribution than metastasis: SDvol, 144 vs 257 (*p* < .001). Areas under the receiver operating characteristic curve was significantly higher with additive DWI than standard MR alone: 0.762 vs 0.953; 0.706 vs 0.950 (*p* < .05) for two readers.

**Conclusions:**

This study suggested that the addition of axial DWI to standard MR imaging can be helpful to diagnose multiple myeloma from metastasis at 3T.

## Introduction

Metastasis and multiple myeloma are common malignant disease involving bone marrow. Metastasis is most common, and appeared in various form of lytic or sclerotic bone lesions. The incidence of multiple myeloma is increasing in recent years [1]. It usually appears as lytic bone lesion in x-ray and CT images, and its MR imaging findings are classified into normal, focal, diffuse, and typical salt and pepper pattern [2–5]. They both present similar MR imaging manifestation and symptoms such as back pain, especially when involving the spine [6]. There were studies using standard MR imaging to differentiate these two diseases involving the spine, but there were some overlaps of MR imaging patterns between multiple myeloma and metastasis [7–8].

Diffusion-weighted imaging (DWI) has been a research topic in various field of musculoskeletal imaging. Previous studies proved that malignant marrow infiltration shows increased water content by destruction of trabecular bone and replacement of marrow fat, thus elevating apparent diffusion coefficient (ADC) value relative to normal background bone marrow [9–11]. In addition, DWI represented better signal to background ratio in detecting malignant bone lesions [12]. There have been various studies on malignant bone lesions including metastasis and multiple myeloma using DWI [10, 11, 13–17]. However, so far comparative studies between the two groups are limited in our knowledge.

Thus, we hypothesized that adding axial DWI to standard MR imaging could help differentiate between multiple myeloma and metastasis. The purpose of our study was to retrospectively determine the value of adding axial DWI to standard MR imaging to differentiate multiple myeloma from metastasis at 3T.

## Methods

### Patient population

**Fig 1:**
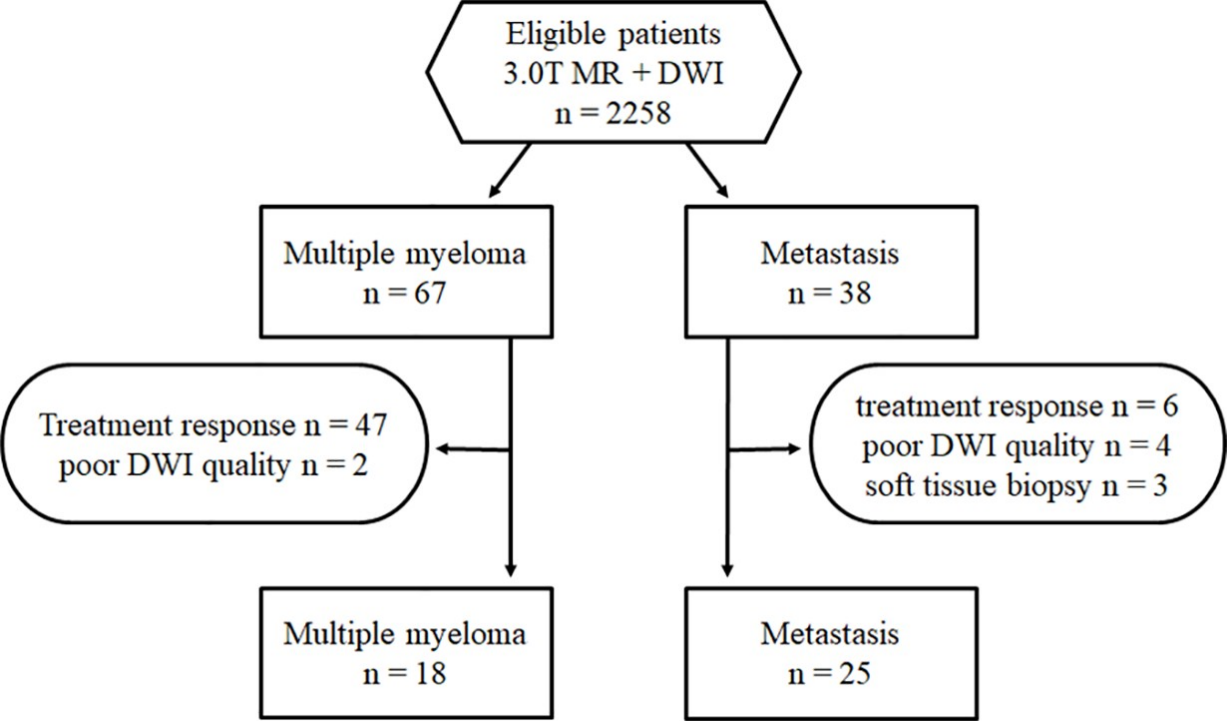
Flow diagram of the study.

This retrospective study was approved by Seoul St. Mary's hospital institutional review board and informed consent was waived. From September 2010 to March 2014, the patients who underwent 3T musculoskeletal MR imaging including DWI were included (Fig 1). A total of 67 patients were diagnosed as multiple myeloma by bone marrow biopsy. Finally, 18 patients with treatment naive multiple myeloma were included in this study except 47 patients who underwent MR after the treatment, and 2 patients who had poor quality DWI for evaluation. Median age was 63 years old (range 41–77 years). The types of multiple myeloma were IgG κ (n = 10), light chain disease κ (n = 3), IgA κ (n = 2), IgA λ (n = 2) and light chain disease λ (n = 1).

There were a total of 35 patients who had confirmed pathology through biopsy or surgery for the bone lesion identified in musculoskeletal MR imaging. Six of these patients were excluded by performing MR after the treatment, and four were excluded due to poor quality of DWI. Finally, 25 patients with untreated metastatic bone lesions were included. Median age was 60 years old (range 29–81 years). The primary cancers were as follows; lung cancer (n = 7), hepatocellular carcinoma (n = 4), renal cell carcinoma (n = 3), stomach cancer (n = 2), prostate cancer (n = 2), ampulla of Vater cancer (n = 1), colon cancer (n = 1), adrenal gland cancer (n = 1), breast cancer (n = 1), ovary cancer (n = 1), melanoma (n = 1) and leiomyosarcoma of bone (n = 1). When these 25 metastatic lesions were classified according to radiographic nature, 14 were osteolytic, 1 was osteoblastic, 1 was mixed appearance, and 9 were not visible on x-ray or CT images.

### MR imaging protocols

All 43 patients in this study underwent MR imaging with a 3T MR scanner (Magnetom Verio, Siemens Healthcare, Erlangen, Germany). The standard MR protocols included sagittal turbo spin echo (TSE) T1- and T2-weighted images axial TSE T1- and T2-weighted sequences. Other sequences were added according to the anatomical location. Chemical shift selective pulse sequences was used for fat suppression. In 40 patients, sagittal or axial fat-suppressed contrast enhanced T1-weighted sequences were also performed. A single-shot echo planar image (SSEPI) prototype pulse sequence was used for DWI in the axial plane before contrast injection. SSEPI MR parameters were as follows: field of view (FOV) 140–370 mm, matrix size 64 x 45–120 x 128, TR 4032–8300 msec, TE 52–85 msec, section thickness 3–9 mm (no gap), number of excitation 1–5, parallel factor 2. ADC map was obtained with two b values of 0 and 800 sec/mm^2^.

### MR imaging analysis

The MR imaging analysis was performed by two radiologists (W.H.J., S.Y.L., with 17 and 5 years of experience in musculoskeletal radiology), who were blind to final diagnosis. They first estimated the diagnosis by assessing standard MR imaging alone. They decided the diagnostic confidence grade on estimated diagnosis with scale of 0 to 4, where 0 = definite metastasis, 1 = probable metastasis, 2 = possible multiple myeloma, 3 = probable multiple myeloma, and 4 = definite multiple myeloma. And then, two readers measured average value of ADC (ADCav) and minimum value of ADC (ADCmin) by manual drawn of regions of interest (ROIs) on the ADC maps in areas corresponding to hyperintense areas on diffusion weighted images with b = 800 sec/mm^2^ on picture archiving and communication system (PACS). They excluded the regions of hemorrhage, necrosis or severe collapse, comparing standard MR imaging side by side. ADCav was obtained by as large as possible drawn ROIs, and ADCmin was chosen among the lowest ADC value of several small ROIs. They measured the ADC values of up to three lesions in each patients in a consensus. Total 72 lesions were included: multiple myeloma (n = 36) and metastasis (n = 36). Finally, they determined the final estimated diagnosis based on the combined information from standard MR imaging and from obtained ADC values, using the diagnostic confidence grade. This process was conducted six weeks later to avoid recall bias. All the images were analyzed in a random order.

Another musculoskeletal radiologist (J.Y.J. with 10 years of experience in musculoskeletal radiology) performed a histogram analysis of the whole tumor volume, using prototype software (OncoTreat; Simens Healthcare, Erlangen, Germany). He knew the location of the lesions which were evaluated by two other radiologists and drew the multiple volumetric ROIs in three dimensional reformatted images of the b = 800 s/mm^2^ using semi-automated technique. ADC values for each voxel within the segmented volume were displayed as histogram. We obtained ADCvol, SDvol, skewness and kurtosis. ADCvol means the mean value of total ADC values from each voxel, and SDvol means the standard deviation of the obtained histogram of whole tumor volume. A total of 46 lesions (24 for multiple myeloma, 22 for metastasis) were evaluated in histogram analysis. The main contributors of measurement failure was small lesions, as the lesions were poorly delineated in reformatted images.

A total of 44 MR imaging studies for 43 patients were analyzed: lumbar spine (n = 17), thoracic spine (n = 9), pelvic bone (n = 5), humerus (n = 3), cervical spine (n = 2), hip (n = 2), sacrum (n = 1), sternum (n = 1), ankle (n = 1), forearm (n = 1), thigh (n = 1), and hand (n = 1).

### Statistical analysis

In quantitative analysis, comparison of the obtained values from DWI between multiple myeloma and metastasis groups was determined by the t test or Mann-Whitney U-test. Interobserver mesurement reliability of two readers for ADC measurement was determined using intraclass correlation coefficient (ICC). An ICC value of less than 0.40 was indicative of poor agreement; 0.40–0.75, fair to good agreement; and more than 0.75, excellent agreement. The receiver operating characteristic (ROC) curve with areas under the curve (AUC) was used to obtain the optimal cut off values for the values that showed significant differences between two groups. Then, the sensitivity, specificity, and accuracy of each cut off values were analyzed.

In qualitative analysis, the sensitivity, specificity, and accuracy of estimated diagnosis by two readers were evaluated, using clinical diagnosis as a gold standard. Diagnostic confidence grade determined by two readers from standard MR image alone (step 1) and from combined DWI with standard MR image (step 2) were evaluated by the ROC curve with AUC. Diagnostic confidence grade 0–1 was considered metastasis, and 2–4 was considered as multiple myeloma. Interobserver variability was evaluated using the kappa value to measure the degree of agreement in diagnosis at each steps. A kappa value of 0.2 or less was regarded as poor agreement, 0.21 to 0.40 as fair, 0.41 to 0.60 as moderate, 0.61 to 0.80 as good, and 0.81 or more as very good. The statistical analysis was performed using commercial software (SPSS, version 19, Chicago, III and MedCalc Software, version 11.3.0.0, Belgium), with *p <* .*05* being considered as significant.

## Result

### Quantitative analysis of DWI

ADCav, ADCmin, of multiple myeloma were significantly lower than those of metastases: ADCav, 754 μm^2^/sec for reader 1 and 733 μm^2^/sec for reader 2 in multiple myeloma vs 1042 μm^2^/sec for reader 1 and 1045 μm^2^/sec for reader 2 in metastasis (*p <* .*001*); ADCmin, 690 μm^2^/sec for each reader in multiple myeloma vs 879 μm^2^/sec for reader 1 and 908 μm^2^/sec for reader 2 in metastasis (*p <* .*001*). Interobserver agreements of ADCav and ADCmin were excellent: ICC = 0.762–0.905 for ADCav, and ICC = 0.844–0.938 for ADCmin between two readers. AUC of ADCav were 0.785 for reader 1 and 0.821 for reader 2 and AUC of ADCmin were 0.717 for reader 1 and 0.776 for reader 2 (*p <* .*05*).

**Fig 2:**
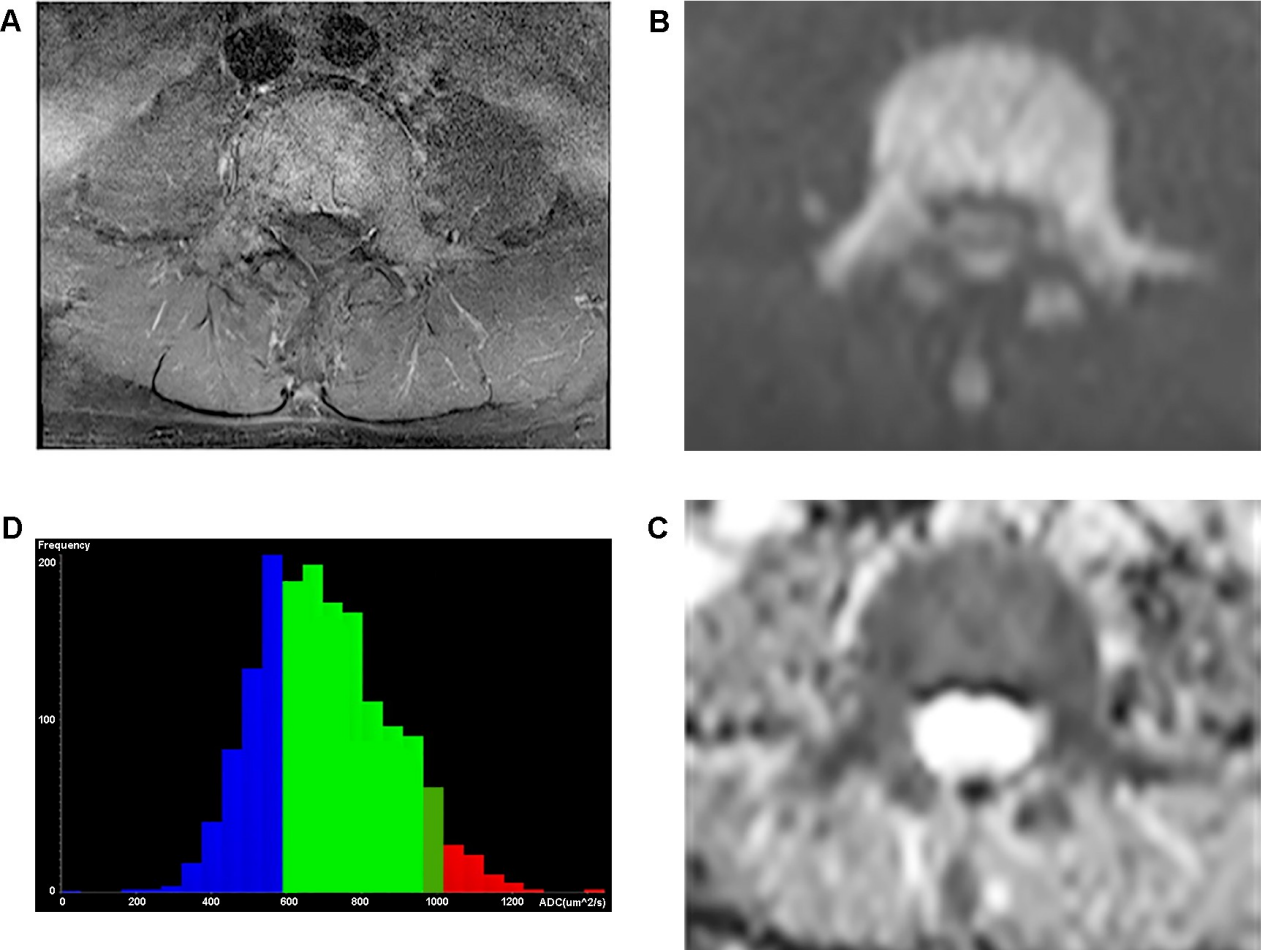
Representative case of multiple myeloma. A 63-year-old man with multiple myeloma. (a) Axial fat-suppressed contrast-enhanced T1-weighted image shows diffuse enhancement in lumbar spine. (b) Axial DW image (b = 800 sec/mm^2^) at the same level reveals diffuse high signals throughout the lesion. (c) Corresponding axial ADC map (b = 0 and 800 sec/mm^2^) shows diffuse low signal intensity. Median value of ADCav was 617 ± 77 μm^2^/sec, ADCmin was 550 ± 75 μm^2^/sec. (d) In the corresponding ADC histogram of whole tumor volume, mean value of ADCvol was 700 μm^2^/sec and SDvol was 178. Interpretation was correct in both readers.

**Fig 3:**
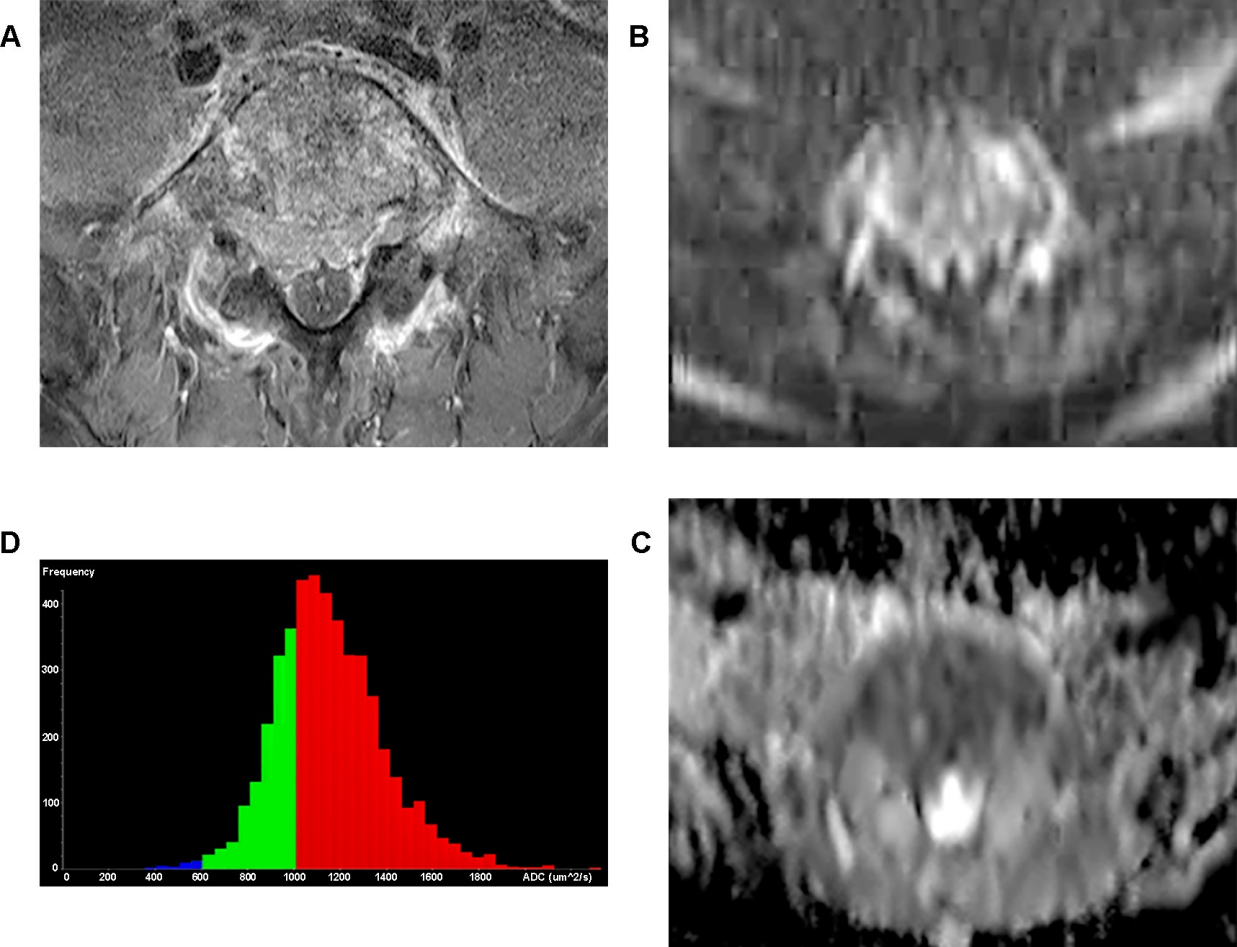
Representative case of metastasis. A 61-year-old man with metastasis from hepatocellular carcinoma. (a) Axial fat suppressed contrast-enhanced T1-weighted image shows pathologic fracture of L5 vertebral body. (b) Axial DW image (b = 800 sec/mm^2^) at the same level reveals hyperintense signals. (c) Corresponding axial ADC map (b = 0 and 800 sec/mm^2^) shows low signal intensity. Median value of ADCav was 1110 ± 150 μm^2^/sec, ADCmin was 1003 ± 57 μm^2^/sec. (d) In the corresponding ADC histogram of whole tumor volume, mean value of ADCvol was 1155 μm^2^/sec and SDvol was 236. Interpretations were correct for both readers.

In histogram analysis for whole tumor volume, ADCvol and SDvol of multiple myeloma was also significantly lower than those of metastases: ADCvol, 761 μm^2^/sec vs 1184 μm^2^/sec (*p <* .*001*); SDvol, 144 vs 257 (*p <* .*001*). Skewness and kurtosis were not different between two groups (*p >* .*05*). (Table 1) (Figs 2 and 3).

**Table 1:** ADC Values with and histogram moments in multiple myeloma and metastasis.

	Multiple myeloma	Metastasis	*p* value
ADCav	752 (619, 849)	1081 (813, 1248)	< .001
ADCmin	704 (587, 773)	835 (709, 1089)	< .001
ADCvol	761 (664, 898)	1184 (1069, 1302)	< .001
SDvol	144 (128, 199)	257 (210, 319)	< .001
Skewness	0.128 (-0.109, 0.482)	0.520 (0.284, 1.00)	0.086
Kurtosis	3.47 (3.09, 5.10)	3.68 (2.93, 5.11)	0.826

Note–Data are median value, and interquartile range in bracket. SD standard deviation. ADC values are in units of μm^2^/sec

Table 2 summarized the cut off value of obtained values that showed a significant difference between two groups. The cutoff values of ADCav, ADCmin, ADCvol, and SDvol were 956 μm^2^/sec (sensitivity 97%, specificity 61%), 765 μm^2^/sec (sensitivity 72%, specificity 72%), 960 μm^2^/sec (sensitivity 83%, specificity 82%), and 192 (sensitivity 71%, specificity 86%), respectively (Table 3). AUCs of ADCav, ADCmin, ADCvol, and SDvol were 0.863 (0.761–0.932), 0.792 (0.681–0.879), 0.805 (0.662–0.907), and 0.833 (0.694–0.927) respectively, without significant difference (*p >* .*05*).

**Table 2:** ADC Cutoff values for differentiating multiple myeloma and metastasis.

	ADCav	ADCmin	ADCvol	SDvol
Cutoff value	956	765	960	192
Sensitivity	97 (35/36)	72 (26/36)	83 (20/24)	71 (17/24)
Specificity	61 (22/36)	72 (26/36)	82 (18/22)	86 (19/22)
Accuracy	79 (57/72)	72 (52/72)	83 (38/46)	78 (36/46)
AUC	0.863 (0.761–0.932)	0.792 (0.681–0.879)	0.805 (0.662–0.907)	0.833 (0.694–0.927)

Note- Data are percentages, with raw data in parentheses. ADC values are in units of μm^2^/sec. SD, standard deviation

**Table 3:** Diagnostic performance in the differentiating of multiple myeloma from metastasis.

	Standard MR imaging alone		Combined DWI and Standard MR imaging	
	Reader 1	Reader 2	Reader 1	Reader 2
Sensitivity	68 (13/19)	74 (14/19)	100 (19/19)	79 (15/19)
Specificity	84 (21/25)	60 (15/25)	92 (23/25)	88 (22/25)
Accuracy	77 (34/44)	66 (29/44)	95 (42/44)	84 (37/44)
AUC	0.772 (0.620–0.884)	0.721 (0.565–0.846)	0.954 (0.844–0.994)	0.886 (0.754–0.962)

Note- AUC, area under the operating characteristic curve. Data are percentages, with raw data in parentheses

### Qualitative analysis of DWI

**Fig 4:**
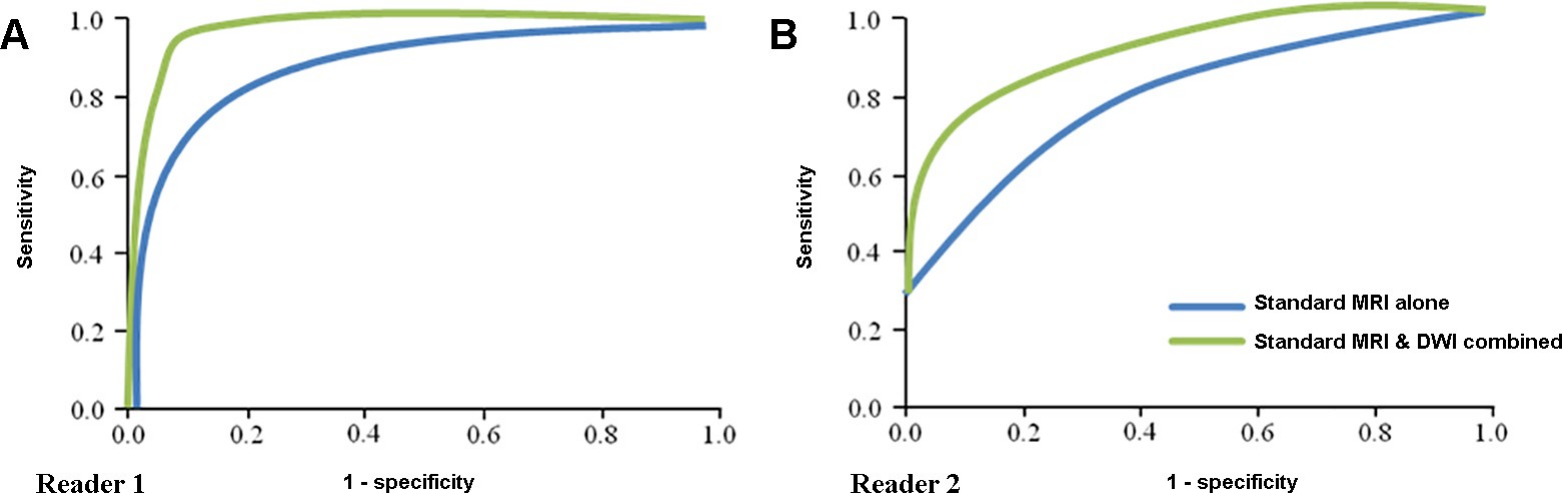
Diagnostic performance in differentiation of multiple myeloma from metastasis. (a) ROC curve of reader 1. Area under the curve (AUC) of standard MR imaging alone was 0.772 and standard MR imaging combined with DWI was 0.954 (*p =* .*002*). (b) ROC curve of reader 2. Area under the curve (AUC) of standard MR imaging alone was 0.721 and standard MR imaging combined with DWI was 0.886 (*p =* .*02*).

With standard MR imaging alone (step 1), sensitivity, specificity and accuracy of reader 1 were 68%, 84% and 77%, and reader 2 were 74%, 60% and 66%, respectively. With standard MR imaging and DWI combined (step 2), the sensitivity, specificity and accuracy were 100%, 92%, and 95% for reader 1, and 79%, 88%, and 84% for reader 2 (Table 3). With addition of DWI to standard MR imaging, diagnostic performance of both readers improved: AUCs improved from 0.772 to 0.954 for reader 1 (*p <* .*05*) and from 0.721 to 0.886 for reader 2 (*p <* .*05*) (Fig 4). Interobserver agreement of the step 1 was moderate (κ = .582) and that of the step 2 was good (κ = .678).

**Fig 5:**
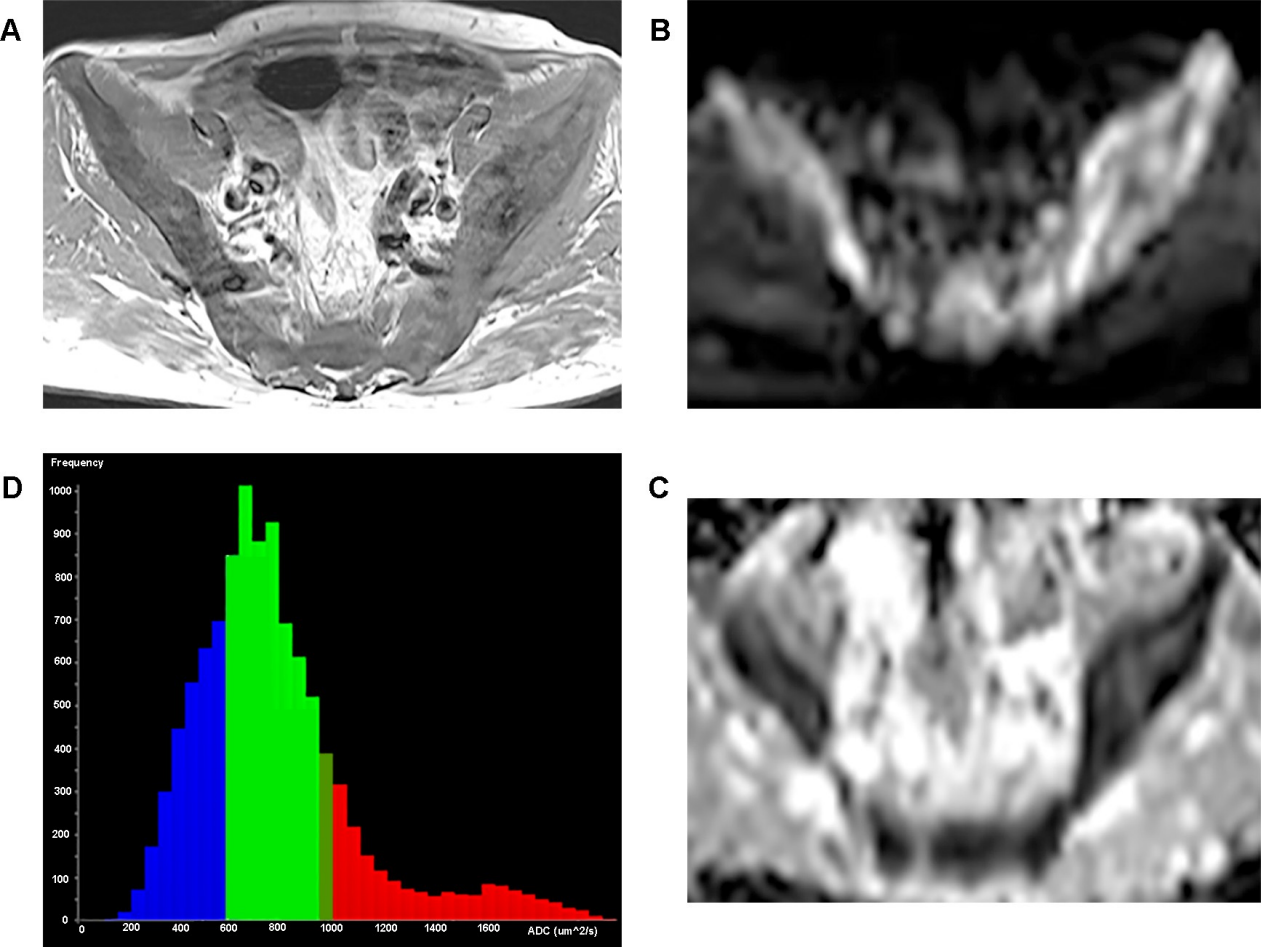
False positive case of prostate cancer. A 77-year-old man with metastasis from prostate cancer (a) Axial T1-weighted image shows diffusely hypointense signals throughout the lesion. (b) Axial DW image (b = 800 sec/mm^2^) at the same level reveals diffuse high signals in the lesion. (c) Corresponding axial ADC map (b = 0 and 800 sec/mm^2^) shows diffuse hypointense signals. Median value of ADCav was 780 ± 143 μm^2^/sec, ADCmin was 731 ± 60 μm^2^/sec. (d) In the corresponding ADC histogram of whole tumor volume, mean value of ADCvol was 767 μm^2^/sec and SDvol was 332. Both readers made a misinterpretation after adding DWI to standard MR imaging.

There was one misinterpreted case of metastatic prostate cancer for both reader 1 and reader 2 (Fig 5). With standard MR imaging, reader 1 made a wrong interpretation and reader 2 made a correct interpretation. However, since the ADC value obtained from DWI was quite low, this case was assessed as multiple myeloma in both reader 1 and 2.

## Discussion

In this study, multiple myeloma showed significantly lower ADC values of ADCav and ADCmin for axial DWI and ADCvol of whole tumor. Moreover, multiple myeloma showed significantly lower SDvol than metastasis on ADC histogram analysis of whole tumor volume.

Padhani et al [15] reported about ADC measurement of 34 myeloma lesions and 69 breast cancer lesions in 1.5 T MR imaging with two *b* values (b = 50, 900 sec/mm^2^). In this study, ADC values of myeloma (875 ± 187 μm^2^/sec) were lower than those of the breast cancer (942 ± 154 μm^2^/sec), although the overlap of ADC values was considerable. Horger et al [16] reported DWI of 12 patients of multiple myeloma with 1.5 T MR imaging, using two b values (b = 50, 800 sec/mm^2^) with mean ADC of 660 ± 150 μm^2^/sec at baseline. Giles et al [17] performed volume based ADC analysis in 18 patients with multiple myeloma with 1.5 T MR imaging, using two b values (b = 50, 800 sec/mm^2^). Mean ADC for whole volume in each patients ranged from 659–971 μm^2^/sec (mean ± SD, 802 ± 89 μm^2^/sec).

In this study, multiple myeloma showed lower ADC values in DWI and narrow distribution in histogram analysis. We speculate that these results may be related to histopathological feature of multiple myeloma. Multiple myeloma is classified as a small cell round tumor of bone. This type of tumor usually represented as uniform, round or oval shaped cells with highly packed arrangement and high nuclear cytoplasmic ratio [18, 19]. This compact arrangement of myeloma cell and associated different molecular pathway limit the free water movement in both extracellular and intracellular space. Therefore, even though the fatty bone marrow is replaced with myeloma cells, there is a certain level of limit on free water movement, so multiple myeloma showed lower ADC value and narrow distribution in histogram.

Lang et el. used DCE-MR to differentiate between multiple myeloma and metastasis [20],and multiple myeloma showed a more aggressive DCE kinetics of wash-out pattern. They interpreted this kinetics as rapid fill up and diffuse back of contrast material, because of the limited cellular space of multiple myeloma.

There was a misinterpreted case of metastatic prostate cancer for both readers. Since the ADC value obtained from DWI was quite low, this case was assessed as multiple myeloma in final decision. Previous studies [5, 9] reported that prostate cancer with diffuse sclerosis showed false negative hypointensity on DWI and lower ADC value. Increased bone trabeculae in sclerotic lesions acts as barriers for free water movements. We used only the MR images for the analysis in this study, and because of this, we could not consider the sclerotic lesion and made a wrong interpretation. Thus, correlation with other imaging modalities such as radiographs or computed tomography scan would be mandatory for correct interpretation.

In this study, we showed that multiple myeloma had lower ADC values than that of metastasis in quantitative analysis. Also, we represented that these quantitative information were helpful to differentiate these two disease in qualitative analysis. Given the different diagnostic and therapeutic approaches of these two diseases, the differential diagnosis using DWI in addition to standard MR imaging may have a clinical significance.

This study has several limitations. First, it is a retrospective study with small study population. Second, metastasis group was composed of heterogeneous histopathology. Third, there may be a selection bias since only patients with pathologically proven bone lesions were included. Because of this, the proportion of multiple myeloma patients in the study population was higher than the general incidence, known as approximately 2.1 per 100,000 persons [1]. Fourth, the ROIs could have contained sclerosis and calcifications that affect ADCs. Finally, only two b values (b = 0 and 800 s/mm^2^) were used for ADC calculation, because these are the most common set of values among the variable MR sequences in our institution. Inclusion of the b value of 0 s/mm^2^ and its perfusion effect of ADC values cannot be ignored, and obtained ADC values can vary between the different DWI protocols.

In conclusion, multiple myeloma showed lower ADC values and standard deviation than metastases on DWI. The addition of axial DWI to a standard MR imaging may helpful to differentiate multiple myeloma from metastases at 3T.

## References

[1] Cowan AJ, Allen C, Barac A, Basaleem H, Bensenor I, Curado MP, et al Global Burden of Multiple Myeloma: A Systematic Analysis for the Global Burden of Disease Study 2016. JAMA oncology 2018. 10.1001/jamaoncol.2018.2128 PMC6143021 29800065

[2] Bauerle T, Hillengass J, Fechtner K, Zechmann CM, Grenacher L, Moelher TM, et al Multiple Myeloma and Monoclonal Gammopathy of Undetermined Significance: Importance of Whole-Body versus Spinal MR Imaging. Radiology 2009;252(2):477–485. 10.1148/radiol.2522081756 19703885 10.1148/radiol.2522081756

[3] Walker R, Barlogie B, Haessler J, Tricot G, Anaissie E, Shaughnessy JD, et al Magnetic Resonance Imaging in Multiple Myeloma: Diagnostic and Clinical Implications. Journal of Clinical Oncology 2007;25(9):1121–1128. 10.1200/JCO.2006.08.5803 17296972 10.1200/JCO.2006.08.5803

[4] Lecouvet FE, Berg BCV, Michaux L, Malghem J, Maldague BE, Jamart J, et al Stage III multiple myeloma: clinical and prognostic value of spinal bone marrow MR imaging. Radiology 1998;209(3):653–660. 10.1148/radiology.209.3.9844655 9844655 10.1148/radiology.209.3.9844655

[5] Stäbler A, Baur A, Bartl R, Munker R, Lamerz R, Reiser MF. Contrast enhancement and quantitative signal analysis in MR imaging of multiple myeloma: assessment of focal and diffuse growth patterns in marrow correlated with biopsies and survival rates. American Journal of Roentgenology 1996;167(4):1029–1036. 10.2214/ajr.167.4.8819407 8819407 10.2214/ajr.167.4.8819407

[6] Resnick D. Skeletal Metastasis In: Resnick D. Bone and Joint Imaging 2_nd_ Ed. Philadelphia, PA: WB Saunders; 1996:1076–1091.

[7] Lee YJ, Jee WH, Ha KY, Lee BY, Kim YS, Kim BS, et al MR Distinction between Multiple Myeloma and Metastasis Involving the Spine. J Korean Radiol Soc 2001;44:229–235.

[8] Kim HJ, Ryu KN, Choi WS, Choi BK, Choi JM, Yoon Y. Spinal Involvement of Hematopoietic Malignancies and Metastsis: Differentiation using MR imaging. Clinical Imaging 1999;23:12–133. 10.1016/s0899-7071(99)00105-9 10416091

[9] Messiou C, deSouza NM. Diffusion Weighted Magnetic Resonance Imaging of metastatic bone disease: A biomarker for treatment response monitoring. Cancer Biomarkers 2010:21–32. 10.3233/CBM-2009-0116 PMC12922841 20164539

[10] Messiou C, Collins DJ, Morgan VA, Desouza NM. Optimising diffusion weighted MRI for imaging metastatic and myeloma bone disease and assessing reproducibility. European radiology 2011;21(8):1713–1718. 10.1007/s00330-011-2116-4 21472473 10.1007/s00330-011-2116-4

[11] Nonomura Y, Yasumoto M, Yoshimura R, Haraguchi K, Ito S, Akashi T, et al Relationship Between Bone Marrow Cellularity and Apparent Diffusion Coefficient. JMRI 2001;13:757–760. 11329198 10.1002/jmri.1105

[12] Pearce T, Philip S, Brown J, Koh DM, Burn PR. Bone metastases from prostate, breast and multiple myeloma: differences in lesion conspicuity at short-tau inversion recovery and diffusion-weighted MRI. Br J Radiol 2012;85(1016):1102–1106. 10.1259/bjr/30649204 22457319 10.1259/bjr/30649204 PMC3587069

[13] Sachpekidis C, Mosebach J, Freitag MT, Wilhelm T, Mai EK, Goldschmidt H, et al Application of 18F-FDG PET and diffusion weighted imaging (DWI) in multiple myeloma: comparison of functional imaging modalities. Am J Nucl Med Mol Imaging 2015;5(5):479–492. 26550539 PMC4620175

[14] Hillengass J, Bauerle T, Bartl R, Andrulis M, McClanahan F, Laun FB, et al Diffusion-weighted imaging for non-invasive and quantitative monitoring of bone marrow infiltration in patients with monoclonal plasma cell disease: a comparative study with histology. British journal of haematology 2011;153(6):721–728. 10.1111/j.1365-2141.2011.08658.x 21517815 10.1111/j.1365-2141.2011.08658.x

[15] Padhani AR, Koh D-M, Collins DJ. Whole-Body Diffusion-weighted MR Imaging in Cancer: Current Status and Research Directions. Radiology 2011;261(3):700–718. 10.1148/radiol.11110474 22095994 10.1148/radiol.11110474

[16] Horger M, Weisel K, Horger W, Mroue A, Fenchel M, Lichy M. Whole-body diffusion-weighted MRI with apparent diffusion coefficient mapping for early response monitoring in multiple myeloma: preliminary results. AJR Am J Roentgenol 2011;196(6):W790–795. 10.2214/AJR.10.5979 21606271 10.2214/AJR.10.5979

[17] Giles SL, deSouza NM, Collins DJ, Morgan VA, West S, Davies FE, et al Assessing myeloma bone disease with whole-body diffusion-weighted imaging: comparison with x-ray skeletal survey by region and relationship with laboratory estimates of disease burden. Clin Radiol 2015;70(6):614–621. 10.1016/j.crad.2015.02.013 25799364 10.1016/j.crad.2015.02.013 PMC4443503

[18] Rajwanshi A, Srinivas R, Upasana G. Malignant small round cell tumors. J Cytol 2009;26(1):1–10. 10.4103/0970-9371.54861 21938141 10.4103/0970-9371.54861 PMC3167982

[19] Li S, Siegal GP. Small Cell Tumors of Bone. Advances in Anatomic Pathology 2010;17(1):1–11. 10.1097/PAP.0b013e3181bb6b9c 20032633 10.1097/PAP.0b013e3181bb6b9c

[20] Lang N, Su MY, Yu HJ, Lin M, Hamamura MJ, Yuan H. Differentiation of myeloma and metastatic cancer in the spine using dynamic contrast-enhanced MRI. Magnetic resonance imaging 2013;31(8):1285–1291 10.1016/j.mri.2012.10.006 23290477 10.1016/j.mri.2012.10.006 PMC3620894

